# Telehealth in Times of COVID-19: Spotlight on Austria

**DOI:** 10.3390/healthcare9030280

**Published:** 2021-03-04

**Authors:** Maria Kletečka-Pulker, Sabine Völkl-Kernstock, Anna Fassl, Elisabeth Klager, Harald Willschke, Sophie Klomfar, Thomas Wochele-Thoma, Eva Schaden, Atanas G. Atanasov

**Affiliations:** 1 Ludwig Boltzmann Institute for Digital Health and Patient Safety, Medical University of Vienna, Spitalgasse 23, 1090 Vienna, Austria; sabine.voelkl-kernstock@meduniwien.ac.at (S.V.-K.); anna.fassl@plattformpatientensicherheit.at (A.F.); Elisabeth.Klager@dhps.lbg.ac.at (E.K.); harald.willschke@dhps.lbg.ac.at (H.W.); Sophie.Klomfar@dhps.lbg.ac.at (S.K.); thomas.wochele-thoma@caritas-wien.at (T.W.-T.); eva.schaden@meduniwien.ac.at (E.S.); 2 Institute for Ethics and Law in Medicine, University of Vienna, Spitalgasse 2-4, 1090 Vienna, Austria; 3 Department of Anaesthesia, Intensive Care Medicine and Pain Medicine, Medical University Vienna, Waehringer Guertel 18-20, 1090 Vienna, Austria; 4 Institute of Genetics and Animal Biotechnology of the Polish Academy of Sciences, Jastrzebiec, 05-552 Magdalenka, Poland; 5 Institute of Neurobiology, Bulgarian Academy of Sciences, 1113 Sofia, Bulgaria; 6 Department of Pharmacognosy, University of Vienna, 1090 Vienna, Austria

**Keywords:** telehealth, telemedicine, COVID-19, healthcare, public health, telemedical tools

## Abstract

Introduction: With the spread of Coronavirus disease 2019 (COVID-19), the world has been experiencing an extraordinary state of emergency. As patients entering a doctor’s practice can potentially infect medical staff and other patients, using digital alternatives wherever possible is a potential solution to avoiding face-to-face encounters. In these conditions, telemedicine is becoming increasingly relevant. Hence, the aim of this study was to examine telemedicine use and gathered experiences during the COVID-19 pandemic in Austria. Materials and Methods: In June 2020, a representative group of Austrian respondents (*n* = 1000) was asked via online survey whether they had contacted a doctor during spring of 2020, and, if so, whether they had used a telemedical method to do so. The survey also reflected gathered experiences and degrees of satisfaction with the use of telemedicine. Results: A third (33%) of those who contacted a doctor during the target period did so using telemedical tools. The majority of those with previous telehealth experience were satisfied with the help they received. Patients commonly used a telephone to contact their doctors. The overall assessment of telemedical aids tended to be positive, with more than half (53%) of those surveyed seeing significant advantages, and a 90% satisfaction rate among the respondents who used telehealth services. Conclusion: The outcomes from this work hint at fairly high acceptance of telemedical communication tools in the studied group of the Austrian population. Based on the high rate of satisfaction among patients who used telehealth, it is expected that the use of telehealth services will increase further in the near future.

## 1. Introduction

In 2020, Coronavirus disease 2019 (COVID-19) captured the world firmly in its grip [1,2]. Routines as we knew them, such as attending a doctor’s practice in person, are changing in light of the global pandemic, and there is a need to encourage new behavior patterns, such as maintenance of physical distancing. Physical distancing during the COVID-19 pandemic has been a result of both voluntary reduction of social contacts and of state-mandated measures, and research in different countries has shown its potential to counteract the spread of the disease [3,4]. On this background, the circumstances are now encouraging the introduction of new methods for conducting medical examinations and consultations. In this situation, telemedicine and e-health are becoming increasingly important, and attracting more attention from both health professionals and medical schools [5,6,7]. It is therefore of relevance to know how patients feel about contemporary healthcare and the healthcare system while they are staying at home; whether and how they have consulted doctors since the curfew and imposing of safety restrictions (during the “first wave” of COVID-19 infections in Austria, starting from the middle of March); whether they have used telemedicine; and, if so, what their experience was.

Telematics, a combination of telecommunications and information technology (IT), is a broader term that describes services provided via a variety of connected IT systems [8,9], while the terms telemedicine, or telehealth, are used to describe a wide range of options for applying information and communications technology (ICT) for medical purposes [10,11,12,13]. Telehealth tools may address consulting specialists, monitoring the status of patients with chronic diseases, or enabling satisfactory medical care in remote areas, as an alternative or supplement to conventional treatment [14,15]. The concept of telemedicine has not been very widespread in Austria before the COVID-19 pandemic, although it offers significant potential for cost-efficient support of patients where actual physical contact is unnecessary or impossible. Studies conducted in Austria between 2015 and 2018 showed that patients prefer continuous personal contact with their treating physicians, with medical care provided via app or telephone, irrespective of location or time, being viewed with skepticism, and with only a few respondents stating that they have had experience with telemedicine [16]. A 2018 worldwide study revealed that telemedicine was also hesitantly adopted in other countries, with the major barriers being technical challenges for the staff, resistance to change, cost, reimbursement issues, the age of the patients, and educational level of the patients [17]. However, in light of the current changes in our daily lives caused by the COVID-19 pandemic, a renewed consideration of telemedicine is appropriate, and better understanding of population attitudes and experiences is of high importance. Thus, this study was designed to survey the prevalence of use and gathered experiences of telehealth application during the COVID-19 pandemic in Austria and to gain insights into the satisfaction levels and attitudes toward the use of telemedicine tools of different demographic groups in the Austrian population.

## 2. Methods

### 2.1. Data Collection

One-thousand computer-assisted web interviews (CAWI) were conducted using closed questions in Austria from 9 to 12 June 2020, addressing the adult population (≥16 years). The questions (in the German language, which is officially spoken in Austria) were defined in collaboration with a specialized demographic research company, Demox Research (https://www.demox-research.com/ (accessed on 28 December 2020)). The questions were in a common language (with the avoidance of specialized terminology) to get a clear message for people with diverse backgrounds. The questions about consulting a physician were formulated in a three-step matrix (Did interviewees had contact to medical services/doctors? How often? In which way?). The sample was drawn from two online panels. Quotas were set to obtain representative data for the Austrian population as a whole. The addresses in the online panel were then randomly selected. The interview questions addressed the healthcare interactions of the respondents for the period after 16 March 2020 (the survey concerned experiences of the respondents for the period from 16 March to 12 June 2020; nationwide restrictions aiming at lowering of COVID-19 spread were implemented in Austria on March 16). In a small number of occasions, some of the 1000 interviewees did not reply to all questions, and for clarity, the number of respondents to the different questions is indicated in the Results section. The data are representative of Austrians older than 16 years of age with access to the Internet.

### 2.2. Data Analysis

Data were analyzed and figures produced with Microsoft Excel (2010 edition) and Adobe Photoshop (CS3 edition). Statistical evaluation of the data was performed with the software package CNT (http://www.wesselhoeft.de/ (accessed on 28 December 2020)), whereby one-tailed Student’s *t*-test was applied and compared groups with *p* < 0.05 were considered to be statistically different.

### 2.3. Ethics Approval

This study is exempt from ethics approval by the Chair of the Ethical Review Board for the Viennese Hospitals in the Vinzenz Holding, since it represents an online survey with voluntary participation, with all data anonymized and the participants de-identified. This exemption is in line with Austrian law and with the principles of the Helsinki Declaration and Europäische Union-Datenschutz Grundverordnung (EU-DSGVO).

## 3. Results

### 3.1. Doctor Consultations

The characteristics of the analyzed population-sample are as follows: 48% of the 1000 respondents were male, and 52% female. The age distribution of the interviewees was fairly balanced (16–29 years: 20%; 30–44 years: 25%; 45–59 years: 26%; 60 years or older: 29%). Of the respondents, 48.5% were employed, 12% were unemployed, 28.5% were retired, and 7.7% were in formal education; 66.9% had a low, 17.3% a medium, and 15.8% a high level of education. To survey the doctor consultations during the target period, the following question was asked: “Since 16th of March, extensive measures to prevent the corona pandemic apply in Austria. Since then (i.e., since March 16 of this year) have you consulted or visited a doctor, regardless of whether they were a general practitioner or a specialist?”. Almost half of the respondents (46%, *n* = 464) of the conducted 1000 interviews had consulted a physician (any kind of consultation, including through physical meeting or by the use of telephone, mobile app, or other telehealth tool) between 16 March and 12 June 2020 during the nationwide Austrian COVID-19 restrictions, while the other half (49%, *n* = 494) did not; 5% either said they did not know, or preferred not to say whether they had consulted a doctor or not during this period. Concerning age, older people (the groups “45–59 years” and “≥60 years”) contacted their physicians significantly (*p* < 0.05) more frequently than young adults (<30 years; see Table 1); 39% of respondents under 30 years of age contacted at least one doctor during this period, while in the older age groups, 45% of those aged 30–44 and 48% of those aged 45–59 had done so; 51% of those aged 60 and above had contacted a physician during this period. Of the 464 respondents who had consulted a doctor, 48% (*n* = 220) were male and 52% (*n* = 243) female; almost two-fifths (39%) had consulted a physician once, and more than half (54%) had consulted a doctor between two and five times, with the remaining 7% having consulted a doctor six or more times.

### 3.2. Form of Contact with the Doctor

Concerning the form of contact, two-thirds (66%) of the 464 respondents had personal contact with their physician in the doctor’s practice, while a quarter (26%) had consulted their doctors chiefly by telephone; 4% communicated with their physician primarily by email; and 3% using chat or video services. Less than two-thirds of respondents in younger age groups visited their doctors in person at the doctor’s practice, preferring to use the telephone, while for respondents aged 60 and above, this figure was 76%.

### 3.3. Satisfaction and Attitude to the Use of Telemedicine

Of the respondents, 155 (48% male, *n* = 75; 51% female, *n* = 79) indicated that they had used telemedicine communication tools to consult a doctor; the majority (65%) were very satisfied with the use of telemedicine. Here, there is a tendency for differences in terms of gender: 7 out of 10 women (70%) were very satisfied with their telemedical encounter, while this was only the case for 6 out of 10 men (59%); however, there was no statistically significant difference between the two gender groups. Interestingly, the two older age groups were especially satisfied (*p* < 0.05) with their telephone or video consultation than those in the younger groups. In the age group below 30 years, 21% of the respondents indicated that they were rather unsatisfied, while just 1% (statistically significant in comparison to the below-30 age group, *p* < 0.05) in the age group 45–59 and 2% (statistically significant in comparison to the below-30 age group, *p* < 0.05) in the age group above 60 years indicated that they were rather unsatisfied. Overall, 24% of the respondents who had a telemedical experience during this time were largely satisfied, and only 7% were largely dissatisfied.

**Figure 1:**
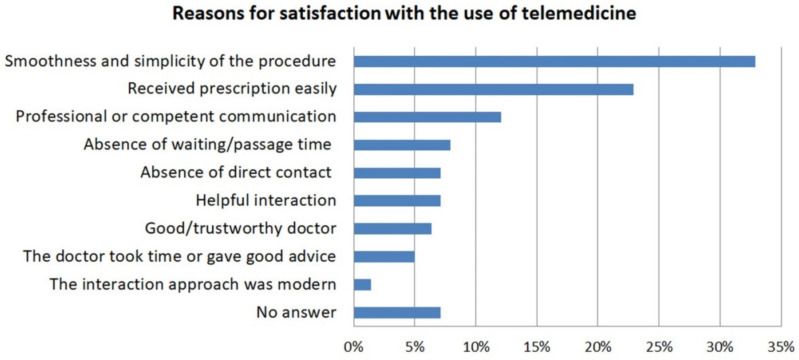
Reasons for satisfaction with the use of telemedicine (*n* = 140).

Among the 140 respondents who had a satisfactory experience with telemedical care during this time, the most frequent reasons for satisfaction were: the smoothness and simplicity of the procedure (32.9%); 22.9% said they had received a certain prescription easily; and 12.1% found the communication to be professional or competent. Other reasons for satisfaction included the absence of waiting/passage time (7.9%); the absence of direct contact (7.1%); the helpful interaction (7.1%); 6.4% indicated that satisfaction was due to a good/trustworthy doctor; 5% were satisfied because the doctor took their time or gave good advice; 1.4% said they were satisfied because of the modern approach (Figure 1). The 12 respondents who received telemedical support but were dissatisfied stated that they did not like online consultation or Zoom conferences, rejected official COVID-19 restrictions in general, or criticized the shortened periods of availability and limited treatment. They were also dissatisfied with the lack of personal contact with the doctor or described the encounter as unorganized and inappropriate. For some, the treatment or communication appeared unprofessional; they felt that patients had been put off; that medical staff had been waiting specifically for COVID-19 patients; and that medical care in general needed to be revised.

A majority of the 155 respondents who communicated with a doctor via telephone or video during the period felt that they had been sufficiently well-understood by the doctor (86%). Of the respondents, 90% of women and 82% of men felt that they had been understood well. The younger respondents aged between 16–29 felt least well-understood, with only 60% of these interviewees stating that they felt sufficiently understood. Of the respondents aged 30–44, 85% shared this impression, while those aged 45–59 (98%) and over 60 (92%) had the highest agreement values when it came to feeling understood. In contrast, one-third (33%) of the respondents who were younger than 30, and 12% of those aged 30–44, had not felt sufficiently understood by their treating physician. The primary reasons for not feeling they had been sufficiently understood were that the respondents felt that the doctor had trivialized their problems in order to avoid personal contact; that specific concerns had been ignored; or that the doctor had not listened. The interviewees stated that the time pressure was noticeable because there were too many patients, and that they had experienced linguistic or acoustic problems.

All respondents—regardless of whether they had had telemedical treatment or not—were asked for their personal attitudes to telemedical applications, video (calling), and emails. The overall picture is rather positive: 53% perceived advantages (39%) or great advantages (14%). In contrast, 35% said they saw disadvantages (30%) or great disadvantages (5%), and 12% of the respondents either did not answer or stated that they did not know whether the use of e-health/telemedicine was more beneficial or disadvantageous.

## 4. Discussion

Our data show that 15.5% of the analyzed Austrian population sample (155 respondents out of 1000) used telehealth solutions between 16 March and 12 June 2020—that is, one-third (33%) of all those who had contact with a doctor during this time were treated remotely using telemedical means. In our survey, we have implemented the term “telehealth” very broadly [18], to cover not only treatment and consultation using other digital tools, but also requesting prescriptions over the telephone, and allowing patients to collect prescription drugs directly from pharmacies without first needing to enter the doctor’s practice. As the telephone is integral to modern life, the inhibition threshold for using the telephone to request prescriptions is relatively low compared to other, more recently developed communication tools.

Our survey revealed that older people, in the age groups of 45–59 years and above 60 years, contacted their doctors significantly more frequently than the representatives of the youngest age group (below 30 years; *p* < 0.05), and these two groups were also significantly (*p* < 0.05) more satisfied with the use of telehealth tools than the youngest age group. When interpreting these data, an important consideration to take into account is that older people generally have more health issues (explaining a need to contact doctors more often) [19], and usually have less experience with new IT technologies (the broader experience of younger people can possibly be a factor linked with higher expectations of the youngest group) [20]. Moreover, important factors playing a role in attitudes of the older patients could be a higher prevalence of diseases hampering mobility in the older patients, and, especially with consideration to the pandemic situation, a greater fear of COVID-19 infection, since this disease has proven more lethal for the older population [21].

The data show that the majority of the respondents (90%) were satisfied or very satisfied with the help or treatment they received via phone, email, chat, or video services, as the process was easy to manage and worked well. These findings are in line with previous research reporting high satisfaction rates with the use of telehealth during the COVID-19 pandemic in different settings, for example in the context of gastroenterology [22] and epilepsy patients [23] in the United States, or among Australian adults [24]. Moreover, high satisfaction with the use of telehealth was also observed in earlier studies prior to the COVID-19 pandemic [25,26].

In contrast to the telephone, specific telemedical apps/tools are only rarely used to communicate with a doctor in Austria, and are therefore not (yet) relevant. This contrasts with the situation, for example, in the United States, where a recent study encompassing 2555 respondents revealed that 50.8% of them used a non-telephone telehealth modality, including 31.9% making use of patient portals and 4.2% of videoconferencing tools [27]. On the background of the lower popularity of more specialized telehealth tools in Austria, taking a “modern approach” just for the sake of being modern apparently seems unimportant, with only 1.4% of respondents who had received and were content with their telemedical care saying they were satisfied because of the modern approach (Figure 1). Additionally, there was little reference to the role of telehealth in virtually eliminating waiting times, as waiting times have been a key factor for respondents in other international studies [28]. Nevertheless, the willingness to use telehealth solutions appears to be on the rise, with 54% of respondents seeing advantages or great advantages to the use of telemedical tools. Consequently, now would be a good time to implement new digital tools as a means of enabling safe digital communication between patients and healthcare providers.

One potential limitation of the methodology used in this study is that it may exclude people who are not experienced in using the Internet and taking online surveys. However, 90% of Austrian households with at least one household member aged between 16 and 74 have access to the internet [29]. Nevertheless, it is likely that persons who already feel comfortable with using the Internet and digital technology to communicate are probably overrepresented in the studied sample. Therefore, generalizations to the total population of Austria based on the results of this study cannot be directly made. Unfortunately, the data in the present study do not allow any statement to be made about patients who needed to contact a doctor during the period in question but did not do so. This could be a worthwhile question for further studies. The acceptance and the use of telehealth in post-pandemic times will be a promising topic for future research, as to date, direct encounters between patients and doctors have been the basis for building a functional patient–doctor relationship [30].

A very important outcome of this study is the notably higher rate of satisfaction with telehealth tools among the people who used them (90%) in comparison with the more modest positive attitude to telehealth in the general population, including people who did not have an experience with telehealth in the study period (53%). This trend indicates that the positive experiences with the use of telehealth tools might even exceed prior expectations, even though there are prevailing positive prior expectations (53%) in the general population.

## 5. Conclusions

In summary, this work profiled attitudes of a 1000-respondent sample of the Austrian population to telemedicine during the COVID-19 pandemic. More than half of the 1000 respondents studied had a positive view of telemedicine and expressed the opinion that it rather offers advantages, and one-third of all 464 respondents who consulted a doctor in the target period had done so using telemedicine tools. Importantly, there was a 90% satisfaction rate among the respondents who used telehealth services, which clearly hints that telemedicine has excellent potential for wider adoption by the Austrian population.

## Figures and Tables

**Table 1:** Consultation of a doctor by age group.

	Yes, I Have Consulted a Doctor (Number of Participants)	Once	2–5 Times	6–10 Times	>10 Times
Totally	46% (464)	39%	54%	4%	3%
<30 years	39% (75)	47%	40%	6%	7%
30–44 years	45% (112)	41%	54%	3%	1%
45–59 years	48% (127) *	34%	57%	4%	4%
≥60 years	51% (150) *	37%	58%	4%	2%
Male gender	48% (220)	37%	53%	6%	3%
Female gender	52% (243)	40%	55%	3%	3%

* Statistically significant difference (*p* < 0.05) compared to the “<30 years” age group.

## Data Availability

The relevant data are contained in the manuscript, and relevant further queries can be addressed to the corresponding authors.
